# Supplementary material for: Experimental protocol to investigate cortical, muscular and body representation alterations in adolescents with idiopathic scoliosis
Source: PLoS One. 2023 Oct 12;18(10):e0292864. doi: 10.1371/journal.pone.0292864 (PMC10569634; doi:10.1371/journal.pone.0292864)
Supplement: S1 Appendix — (PDF) [file pone.0292864.s001.pdf]

S1 Appendix

Guidelines to reproduce the experimental protocol

Resting state recording. Subjects will be equipped with two triplets of passive retro-reflective markers, placed one on each foot at the level of the insertion aspect of the achilles tendon in the calcaneus, the dorsal aspect of the fifth and first metatarsal head, to measure the support base during tasks. Subjects will be asked to stand upright on a force platform, and the position of their feet will be marked with adhesive tape to ensure consistency of foot positioning among repetitions.

Maximum Voluntary Contractions (MVC). Guidelines to find the location of the surface electromyography (sEMG) electrodes and instructions to record MVC from each muscle are presented in Table A 1

Target reaching. First, the physiotherapist - the same for all subjects - will measure the following distances with a soft meter: (a) acromion process - ulnar epicondyle (olecranon); (b) ulnar epicondyle - middle finger pad. The arm length of the subjects will be the sum of (a) and (b). During the measurement, subjects will stand leaning against the wall. Second, a poster goniometer (90-cm diameter) representation will be placed on a hard surface (i.e., a wall or a rigid panel) at a distance equal to 10% more than the subjects' arms. Its center will be aligned with the chest of the subjects. Ten targets, differing in geometrical shape and color, are depicted at 75% of the length of the goniometer rays, oriented according to the following inclinations: 0°, 40°, 70°, 110°, 140°, 180°, 220°, 250°, 290° and 320°. Additional shapes are randomly distributed on the surface of the goniometer to slow the learning process of the subjects. Third, a board cluster consisting of four retro-reflective markers will be placed on the hard surface where the poster is secured to define a local reference frame solid with the poster itself: i.e., in the upper left corner. Subjects will be asked to stand in front of the board, with one arm resting along the body and the other at the chest. Subjects will observe the experimenter, standing next to them, touching (for 3 s) one of 10 ($i = 1, 2, \ldots, 10$) targets (represented in Fig 2A) on the board (eT_i). Subjects will then be instructed to reach the same point (sT_i) before returning to the starting position with the hand at the chest, keeping their eyes closed. The 10 targets will be presented to the subjects in a randomized order. The same task will be repeated both with the dominant and with the non-dominant hand. The coordinates selected by the experimenter (eT_i) and the coordinates selected by the subjects (sT_i) will be measured using the stereophotogrammetric system tracking the 3D position in the space of two reflective markers placed on the index fingers of the experimenter and the subjects, respectively. The coordinates eT_i and sT_i will be transformed into the local coordinate frame of the board, using the board cluster. The absolute Euclidean distance between the local x-y coordinates (i.e., defining the board plane) of the two locations will be calculated for each target as a measure of task execution accuracy.

Forearm bisection task. Subjects will be seated blindfolded on a chair with their dominant arm resting on a table with their elbow flexed at 90° and their hand facing inward. Their nondominant arm will be resting by their side. A separator (15 cm in

Table A 1. Placement of sEMG electrodes and recording of MVCs.

Muscle	Placement	MVC
Latissimus Dorsi (LD)	Over the muscle belly at the T12 level and along a line connecting the most superior point of the posterior axillary fold and the S2 spinous process [1–3].	Prone lying with the side being tested aligned with the edge of the table. Subjects flex their elbow and extend their humerus parallel to the trunk. Manual resistance is provided at the distal humerus. Stabilization is provided to the ipsilateral scapula and contralateral pelvis [4].
Erector Spinae (ES)	Horizontally aligned with the L3-4 interspace, 4 cm lateral to midline [5, 6].	Prone lying with the subjects' shoulder at the edge of the table and hands at sides. Stabilization is provided at the pelvis and ankles. Subjects extend the lumbar spine until the entire trunk is raised from the table [7].
Rectus Abdominis (RA)	Centre of the second uppermost section of the muscle belly [8].	Supine lying with knees and hips both bent to approximately 90° and hands at sides. Stabilization is provided at the legs, braced by the experimenter, and the thorax is strapped down with a belt. Subjects flex the lower trunk in the sagittal plane against manual resistance provided at the shoulder.
Abdominal External oblique (AEO)	Approximately 3 cm anterior to and mid-way along a line drawn from the lateral pelvic crest to the lateral lower ribcage [8].	Side lying position with the knees bent and strapped with a belt, and thorax and arms were manually braced by the experimenter. Subjects attempt to side bend the upper trunk in the frontal plane while manual resistance is provided.
Abdominal Internal Oblique (AIO)	Halfway between the anterior superior iliac spine of the pelvis and the midline, just superior to the inguinal ligament [8].	Right and left side bridge position while maximally resisted downward pressure on the pelvis is applied by the experimenter.

height) will be placed along the forearm of the dominant arm (see Fig 2B). Subjects will be asked to repeatedly move the index finger of their nondominant hand to touch the separator at the level of the midpoint of their dominant forearm (Fig 2B). The separator will avoid tactile feedback. Subjects will be required to return the nondominant arm to the starting position at the end of each trial and to wait for a start signal from the experimenter to perform the movement. The task will be repeated 10 times consecutively. The midpoint of the subjects' arm (target) will be calculated as the midpoint (aT) of the line connecting two retro-reflective markers, one placed on the lateral epicondyle of the humerus and one placed on the radius-styloid process of the wrist of the dominant arm. A third marker will be placed on the posterior aspect of the

forearm, on the midline joining the wrist epicondyles, to build a forearm cluster and define a local coordinate frame. The 3D coordinates in the index-pointed location space will be measured by tracking a reflective marker placed on the index finger of the subjects (sT_i). The distance between the target position (aT) and the position of the subject-selected location (sT_i) along the line connecting the epicondyle and the radius-styloid process of the wrist will be calculated for each repetition as a measure of the accuracy of task execution.

Cognitive and behavioral assessment.

Table A 2. Body Uneasiness Test (BUT) scoring instructions [9].

BUT-A (page 1: items 1-34)	BUT-B (page 2: items 1-37)
**Global measure**	**Global measure**
Global Severity Index: **GSI** = Average rating of all 34 items of BUT-A	*Positive Symptom Total*: **PST** = Number of symptoms rated higher than zero
	Positive Symptom Distress Index: **PSDI** = Average rating of those items constituting the Positive Symptom Total
**Subscales**	**Subscales**
Weight Phobia: **WP** = Average rating of items: 9; 10; 18; 21; 24; 31; 32; 33	*Mouth*: **BUT-B I** = Average rating of items: 7; 8; 9; 10; 11; 12
Body Image Concerns: **BIC** = Average rating of items: 3; 4; 6; 12; 15; 22; 23; 25; 34	*Face Shape*: **BUT-B II** = Average rating of items: 2; 3; 6; 13; 14; 15
Avoidance: **A** = Average rating of items: 5; 8; 13; 16; 19; 30	*Thighs*: **BUT-B III** = Average rating of items: 24; 25; 28; 29; 30
Compulsive Self-Monitoring: **CSM** = Average rating of items: 1; 11; 17; 20; 27	*Legs*: **BUT-B IV** = Average rating of items: 1; 21; 31; 32; 33
Depersonalization: **D** = Average rating of items: 2; 7; 14; 26; 28; 29	*Arms*: **BUT-B V** = Average rating of items: 19; 20; 22; 23; 26
	Moustache: **BUT-B VI** = Average rating of items: 16; 17; 18
	Skin: **BUT-B VII** = Average rating of items: 4; 5
	Blushing: **BUT-B VIII** = Average rating of items: 27; 34; 35; 36; 37

Computerized body self-recognition task. The *Computerized body self-recognition task* consists of random visualization of 96 images (72 non-self-images and 24 self-images) and will be preceded by a training consisting of 2 images to familiarize with the mechanism (Fig A 1). To make the subjects' focus on the anatomical characteristics of the body part rather than its position in the space, the body part in the upper and lower images could be captured in a position equal to each other but different from the central template. The presentation and randomization of

the images will be controlled with PsychoPy (v2022.1.4). The photographs for the *Computerized body self-recognition task* will be taken by the same physician for all subjects during the first medical examination with a digital camera. Subjects, turning their back to the physiatrist, will remove upper clothing only for the photographs of their shoulders and back). The photographs will be modified to images with a uniform white background (Fig A 2) by means of a background eraser software (e.g., removebg, Kaleido AI GmbH).

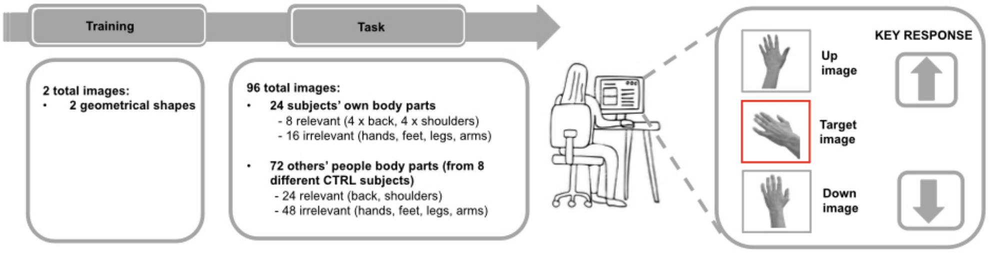

Fig A 1. Computerized body self-recognition task. From left to right, a description of the test and an example of presentation of body images and possible response actions are shown.

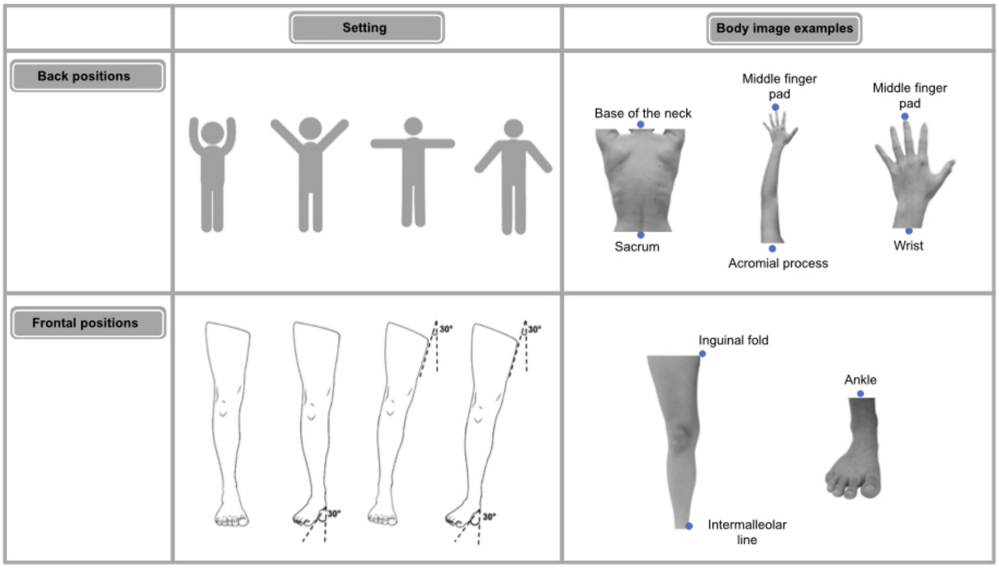

Fig A 2. Body images for the computerized body self-recognition task In the second column, the subjects' positions during photograph acquisition are visualized. In the third column, some examples of relevant (i.e., back, shoulders) and irrelevant (i.e., foot, hand, leg, arm) body images are reported. The blue points are reported only as guidelines for the experimenters.

References

1. Ng JKF, Parnianpour M, Richardson CA, Kippers V. Effect of fatigue on torque output and electromyographic measures of trunk muscles during isometric axial

rotation. Archives of physical medicine and rehabilitation. 2003;84(3):374–381.

2. Ng JKF, Parnianpour M, Kippers V, Richardson CA. Reliability of electromyographic and torque measures during isometric axial rotation exertions of the trunk. Clinical Neurophysiology. 2003;114(12):2355–2361.
3. Fan JZ, Liu X, Ni GX. Angular velocity affects trunk muscle strength and EMG activation during isokinetic axial rotation. BioMed research international. 2014;2014.
4. Park Sy, Yoo Wg. Comparison of exercises inducing maximum voluntary isometric contraction for the latissimus dorsi using surface electromyography. Journal of Electromyography and Kinesiology. 2013;23(5):1106–1110.
5. Raschke U, Chaffin D. Trunk and hip muscle recruitment in response to external anterior lumbosacral shear and moment loads. Clinical Biomechanics. 1996;11(3):145–152.
6. Kumar S. EMG in rotation–flexion of the torso. Journal of Electromyography and Kinesiology. 2010;20(6):1146–1154.
7. Hislop H, Avers D, Brown M. Daniels and Worthingham's muscle Testing-E-Book: Techniques of manual examination and performance testing. Elsevier Health Sciences; 2013.
8. Vera-Garcia FJ, Moreside JM, McGill SM. MVC techniques to normalize trunk muscle EMG in healthy women. Journal of electromyography and kinesiology. 2010;20(1):10–16.
9. Cuzzolaro M, Vetrone G, Marano G, Garfinkel P. The Body Uneasiness Test (BUT): development and validation of a new body image assessment scale. Eating and Weight Disorders-Studies on Anorexia, Bulimia and Obesity. 2006;11:1–13.
