# Supplementary material for: Experimental protocol to investigate cortical, muscular and body representation alterations in adolescents with idiopathic scoliosis
Source: PLoS One. 2023 Oct 12;18(10):e0292864. doi: 10.1371/journal.pone.0292864 (PMC10569634; doi:10.1371/journal.pone.0292864)
Supplement: Attachment — Submitted filename: Response_to_Reviewers_40822.pdf [file pone.0292864.s002.pdf]

DIPARTIMENTO DI NEUROSCIENZE
Direttore: Prof. Raffaele De Caro

via Belzoni, 160
35121 Padova– Italy
Tel. +39 049 827 2025/2036
e-mail: direzione.neuroscienze@unipd.it
PEC: dipartimento.neuroscienze@pec.unipd.it

CF 80006480281

P.IVA 00742430283

Padova, August 4, 2023

Response to reviewers for manuscript: "Experimental protocol to investigate cortical, muscular and body representation alterations in adolescents with idiopathic scoliosis"

Dear Editor,

We would like to thank the Reviewers for the careful evaluation of our work, as well as for the constructive comments and suggestions they provided which improved and enriched our work. We have considered each of the comments and revised our manuscript accordingly.

Below we provide a point-by-point response (text in blue) to each of the comments (text in black). The related changes in the manuscript are in red.

The manuscript has been substantially revised following the suggestions of the Reviewers. Specifically, thank you to the Reviewers' feedback:

- We clarified the rationale behind the aim of the study: *The primary aim of this experimental protocol is to quantitatively investigate alterations in the body representation in AIS, and to quantitatively and objectively track the modifications in the sensorimotor representation of the body due to the treatment*;
- We clarified the rationale behind the methodologies employed: *1) The forearm bisection task is a simple and common test widely used in clinical and research fields to investigate the body schema; 2) The target reaching forces the subjects to perform forward trunk movements to investigate if alterations of body schema can modify the planning of the movement within the action space immediately surrounding the body.* The instrumentation of these two tasks will allow us to quantitatively track the changes in body schema.

We hope that the manuscript will be now suitable for publication in *PLOS ONE*.

On behalf of all authors,

Kind regards,

Maria Rubega, Ph.D.

Editor Comment to the Author

Authors have presented a good quality paper, however, there are still points that have to be clarified and require a major revision of the manuscript. In particular, Authors have to clarify the rationale behind the aim of the study and of the methodologies employed as also suggested by the reviewers.

Review Comments to the Author

Reviewer #1:

I have the following comments regarding the protocol:

1) Sample size appears relatively small, especially if future statistical analyses requiring further subgrouping. I understand that the sample size proposed was based on the difference between control and AIS detected in another study as cited. What if the difference detected is smaller than what was found in that study? Please, provide plans of power analyses and whether there is a need of review of sample size as an interim measure for this study.

1) We agree with the Reviewer. As suggested, power statistical analyses will be repeated after having recorded data at T0 as an additional tool to review the sample size required in order to detect the effect of AIS in the parameters recorded in the experimental protocol. If necessary, the sample size will be enlarged.

Please, see lines 191-193, page 6:

Power statistical analyses will be repeated after having recorded data at T0 as an additional tool to review the required sample size. If necessary, the sample size will be enlarged.

2) Sexual maturity and Risser sign were collected as maturity measures - Risser sign has been known for its insensitivity and inaccuracy of its use in AIS. I would suggest the authors to consider additional skeletal maturity measure that will be available for assessment on whole spine radiographs such as the Proximal Femur Maturity Index (PFMI).

2) We thank the Reviewer for the suggestion.

Actually, some authors report a limited sensitivity of Risser staging during peak growth velocity and lower interobserver reliability. However, the most recent guidelines on scoliosis management (SOSORT 2016) state the Risser sign as the marker of skeletal maturity that is most used in decision-making in the treatment of adolescent idiopathic scoliosis.

We agree with the Reviewer about PFMI. It is a newly developed marker of skeletal maturity that may aid in clinical decision making. We will add it in our protocol (see Fig1). Beyond that, there is currently a small amount of literature indicating the reliability of this classification scheme, which needs to be evaluated through additional studies. For the above reasons, we will use both (i.e., Risser and PFMI).

Please, see Figure 1, we added the Proximal Femur Maturity Index.

3) The duration of 80 minutes of tests appear quite long for adolescents, plus the additional Protocol feasibility assessment at the end of the experimental session - please consider

adopting the separate sessions approach as proposed, or to condense the experimental session.

3) We agree with the Reviewer.

A risk of the proposed experimental protocol is boredom and loss of interest and compliance due to its duration and complexity. If necessary, we can divide the protocol into three separate sessions on three different days. Block (i), blocks (iii-iv), and block (v) of the protocol are independent and investigate different research questions.

Please, see lines 488-492 , page 12:

A risk of the proposed experimental protocol is boredom and loss of interest and compliance due to its duration (circa 80 min) and complexity. If necessary, the protocol can be divided into three separate sessions on three different days. Block (i), blocks (iii-iv), and block (v) of the protocol are independent and investigate different research questions.

Up to now, two subjects with untreated AIS have undergone the experimental protocol (at T0) presented in the manuscript in one day.

4) Regarding brace-wear compliance - is there an objective measure of compliance such as by thermal sensor in the brace? Brace-wear compliance can have an impact on the muscles and thus can be affecting the study results.
Also wondering if there is any measure to take into account brace correction rate and correct brace-wear at how many hours in a day (%).

4) Thermal sensors in the brace are not commonly used in clinical practice because it implies an additional (not-negligible) cost for families. An X-ray will be taken before and after wearing the brace. To evaluate the in-brace correction, the initial correction rate, i.e. (Cobb angle after brace wearing / Cobb angle before brace wearing) * 100, will be estimated. We added this initial correction rate (ICR) in the methods section.

To our knowledge, the strength/endurance of the back muscles is not affected by the use of a brace in adolescents with idiopathic scoliosis (e.g., in the work of Pikulska et al., 2021, the authors reported no impact of 6-month brace wear on muscle strength or endurance in back muscle function in adolescents treated with a rigid brace for idiopathic scoliosis).

Rather, proprioception could be influenced by prolonged brace use. Quantitatively estimating the effect in proprioception is one of our primary goals.

In-brace correction will allow us to evaluate how strongly the brace itself can modify the curve. Beyond that, the objective of the study is to analyze when the proprioceptive input of the brace can shape the body perception in general.

Our team has developed a smartphone application in which girls with AIS and/or their parents can record the number of hours/day of brace-wear. We are aware that it is not a reliable measure like the one proposed by the Reviewer, however it allows us to have an estimate of the wearing time of the brace.

Please see lines 237-239, page 7:

Based on the radiological image, the physiatrist will also collect other relevant clinical measures, i.e., the initial correction rate (ICR), the side and anatomical site of the convexity of the scoliosis, trunk asymmetries, shoulders, and hips inclination.

References

Pikulska, D., et al. "Back muscle function in adolescent girls treated with a rigid brace for idiopathic scoliosis: no impact of 6-month brace wear on muscle strength or endurance." Stud Health Technol Inform 280 (2021): 168-73.

5) There has always been an issue regarding how accurate patients can perform scoliosis-specific exercise exactly on their own and the accuracy of exercise duration reports. Again this aspect of the exercise can potentially affect study results. Any thoughts by the authors on that?

5) We thank the Reviewer for the observation.

Participants will be monitored in their performance in scoliosis-specific exercise at least once a week by a physiotherapist expert in scoliosis. Furthermore, using a dedicated smartphone application developed by our group, we will monitor compliance with the exercise programme.

Please, see lines 155-158, page 5:

After this evaluation (T0), if the adolescent shows low-grade scoliosis, an expert physiotherapist gives her corrective postural exercises to perform daily (30 min / day) until growth is complete. Participants' performance in scoliosis-specific exercise will be monitored by the physiotherapist at least once a week.

6) This study emphasizes on quantifying EEG and sEMG signals during the progression of AIS - the authors should define how they evaluate and define curve progression in other to achieve the above.

6) We thank the Reviewer for the observation.

We have now highlighted that a full spine in orthostatism radiography will be performed at T2 to track the progression of scoliosis. The progression of AIS will be defined as a worsening of more than 5° Cobb in the magnitude of the curve.

Please, see lines 242-244, page 7:

The progression of AIS will be defined as a worsening of more than 5° Cobb in the magnitude of the curve during the duration of the study (approximately one year).

Reviewer #2:

This study protocol is interesting as several measures will permit assessing different neural mechanisms across time. However, the authors are not sufficiently clear about what they will analyze, and they do not present the rationale for proposing these tasks.

We thank the Reviewer for taking the time to carefully revise our work and to let us clearly identify and justify our research questions.

For sake of readability, in the first version of our work, we reported many details about the chosen tasks in the Supplementary Material.
Now, we provided a clear rationale for the proposed study in the main manuscript as requested by the Reviewer. Moreover, we differently organized the Introduction to clarify the rationale of the study. First, we introduced the concept of AIS and we described the treatment. Second, we reported studies aimed to investigate CNS in AIS. Third, we reported studies about human motion analysis in AIS. Fourth, we introduced the key concepts of body schema and body image. Fifth, we reported our aim and how to achieve it.

Here are my comments concerning this study protocol.

P2 – L36: Should mention/explain the task rather than simply state it is a simple task.

We agree with the Reviewer. We added details on the motor task.

Please, see lines 38-39, page 2:

Abnormal patterns were detected in the motor network during the execution of a simple motor task which consisted in opening and closing one's fist.

P3- L60: I am surprised by this statement as several studies reported balance control impairment in AIS compared to controls during sensory manipulation while participants stood upright. I encourage the authors to scan the literature.

We thank the Reviewer for allowing us to clarify this aspect.

We agree with the Reviewer. Balance control measured through force plates revealed no differences between girls with AIS and CTRL in a standing upright position (Formaggio et al., 2022, Fortin et al., 2019, Pialasse et al., 2015, Byl et al., 1993). Differences between girls with AIS and CTRL have been detected when the experimenters asked the participants to stand upright while being engaged in a second task (i.e., applying vibration to a muscle tendon, asking the subjects to raise the arms up with eyes closed, or during galvanic vestibular stimulation, (Fortin et al. 2019, Formaggio et al. 2022, Pialasse et al., 2015). These differences during dual tasks were also detected in brain activity (Formaggio et al., 2022, Fortin et al., 2019).

Please see lines 62-67, page 3:

Balance control measured through force plates revealed no differences between girls with AIS and CTRL in a standing upright position [3, 14, 16, 17]. Differences between girls with AIS and CTRL have been detected when the experimenters asked the participants to stand upright while engaged in a second task (i.e., applying vibration to a muscle tendon, asking the subjects to raise their arms with their eyes closed, or during galvanic vestibular stimulation [3, 14, 16].

References

Byl, NN., Gray, JM. (1993). Complex balance reactions in different sensory conditions: adolescents with and without idiopathic scoliosis. J Orthop Res., 11(2):215-27.

Formaggio, E., Bertuccelli, M., Rubega, M., Di Marco, R., Cantele, F., Gottardello, F., ... & Masiero, S. (2022). Brain oscillatory activity in adolescent idiopathic scoliosis. *Scientific Reports*, *12*(1), 17266.

Fortin, C., Pialasse, J. P., Knoth, I. S., Lippé, S., Duclos, C., & Simoneau, M. (2019). Cortical dynamics of sensorimotor information processing associated with balance control in adolescents with and without idiopathic scoliosis. *Clinical Neurophysiology*, *130*(10), 1752-1761.

Pialasse, J. P., Descarreaux, M., Mercier, P., Blouin, J., & Simoneau, M. (2015). The vestibular-evoked postural response of adolescents with idiopathic scoliosis is altered. PLoS One, 10(11), e0143124.

P3 – L93: The authors should explain what the goal of these tasks is. How will the results of these tasks allow the authors to identify the neural mechanisms related to curve progression or can trigger the Stoke's Vicious Cycle Pathogenesis?

We thank the Reviewer for the suggestion.

The goal of the *forearm bisection task* and the *target reaching* has been clarified in the new version of the manuscript. These tasks aim to investigate the body schema in adolescents with idiopathic scoliosis that has been proposed to be parallel to the development of spinal deformity. Representational changes in body schema may reflect altered activation of the sensorimotor network. The recording of the EEG signal during the execution of these motor tasks at T0, T1 and T2 will allow us to investigate the activation of the sensorimotor cortex where the body schema is 'dynamically' represented, before and during AIS treatment.

Please see lines 84-94, page 3:

Body schema is generally assessed using self-reported assessment scales [19]. We propose two ad-hoc tasks to monitor it in a longitudinal study. (i) The *forearm bisection task* is a simple and common test widely used in clinical and research fields to investigate the body schema [23]. It requires the subjects to point to the perceived midpoint of their forearm. Since AIS significantly changes the structure of the adolescent spine, thorax, and trunk, we include an innovative task that involves the movement of the trunk. (ii) The *target reaching* forces the subjects to perform forward trunk movements. It aims to investigate the body schema in adolescent girls with AIS and to determine if alterations in the body schema can modify the planning of movement within the action space immediately surrounding the body, also known as the peripersonal space.

Please see lines 98-101, page 4:

The EEG signal allows us to quantitatively record the activation of the sensorimotor cortex where the body schema is 'dynamically' represented. In fact, the representation of the body schema is plastic and can vary [23].

P4 – L97: I encourage the authors to be more specific here. In the present form it is unclear whether the authors have specific hypotheses. The dimension for exploring neurophysiological and muscular changes is infinite! How tracking will be realized?

We agree with the Reviewer.

The hypothesis and the aim of the study were not clearly expressed. We aim to quantitatively investigate alterations in the body schema in adolescents with idiopathic scoliosis. Body schema alterations reflect sensorimotor representation of the body used to plan and execute movements and have been proposed to be parallel to the development of spinal deformity. Investigating the body schema alterations in AIS, before and after 6-months of therapy, may help us to better understand pathophysiology and etiology of scoliosis. To reach our aim, we propose two ad-hoc tasks: the forearm bisection task and the target reaching. During these two tasks, the performance of the subjects will be quantitatively assessed using EEG, marker-based stereophotogrammetry, IMUs, sEMG, and force plates. In particular, EEG signals will be collected to examine altered activation of the sensorimotor network related to possible representational changes in body schema.

We rephrased the aim of the study at the end of the Introduction paragraph.

A detailed description of how the subjects movements will be tracked during the experiment and how task execution accuracy will be estimated are provided in S1 Appendix.

For the *target reaching,* please, see lines 30-37, page 1, S1 Appendix.

The coordinates selected by the experimenter (eT_i) and the coordinates selected by the subjects (sT_i) will be measured using the stereophotogrammetric system tracking the 3D position in the space of two reflective markers placed on the index fingers of the experimenter and the subjects, respectively. The coordinates eT_i and sT_i will be transformed into the local coordinate frame of the board, using the board cluster. The absolute Euclidean distance between the local x-y coordinates (i.e., defining the board plane) of the two locations will be calculated for each target as a measure of task execution accuracy.

For the *forearm bisection task,* please, see lines 47-57, pages 2-3, S1 Appendix.

The midpoint of the subjects' arm (target) will be calculated as the midpoint (aT) of the line connecting two retro-reflective markers, one placed on the lateral epicondyle of the humerus and one placed on the radius-styloid process of the wrist of the dominant arm. A third marker will be placed on the posterior aspect of the forearm, on the midline joining the wrist epicondyles, to build a forearm cluster and define a local coordinate frame. The 3D coordinates in the index-pointed location space will be measured by tracking a reflective marker placed on the index finger of the subjects (sT_i). The distance between the target position (aT) and the position of the subject-selected location (sT_i) along the line connecting the epicondyle and the radius-styloid process of the wrist will be calculated for each repetition as a measure of the accuracy of task execution.

P4 – L104: What treatment the authors are referring to? In addition, will all participants have

the same treatment? If not, how will the authors interpret changes across time if the treatment differs among the participants?

We thank the Reviewer for having raised this issue.

We added further details on the treatment that adolescents with idiopathic scoliosis will undergo in the Experimental Design paragraph. The set of exercises will be taught and adapted from the physiotherapist considering each participant's characteristics and clinical assessment, but all the exercises proposed will share the same aims (i.e., to improve postural awareness, proprioception, and ability to dissociate the movements of the lumbar spine from the thoracic one in the different planes of space).

Please, see lines 155-163, page 5:

After this evaluation (T0), if the adolescent shows low-grade scoliosis, an expert physiotherapist gives her corrective postural exercises to perform daily (30 min / day) until growth is complete. Participants' performance in scoliosis-specific exercise will be monitored by the physiotherapist at least once a week. The set of exercises is believed to improve postural awareness, proprioception, and ability to dissociate the movements of the lumbar spine from the thoracic one in the different planes of space. Treatment will be planned in the individual patient, based on the dysfunction detected in the clinical assessment. When scoliosis exceeds the 20° Cobb and there is evidence of progression risk, a rigid partial (18 hours/day) or full-time brace (23 hours/day) is also prescribed.

P4 – L105: I am confused; will the authors offer participants psychological support or cognitive behavior therapies?

We thank the Reviewer for letting us clarify this issue.

We will not offer participants psychological support. We would like to propose a quantitative test to detect the need of psychological aid and collect evidence to support this hypothesis. We removed this misunderstanding sentence from the manuscript.

P4 – L108: I agree with these hypotheses, as several previous studies have reported these changes between controls and AIS. However, how will these results bring novel knowledge and allow the authors to identify biomarkers causing curve progression or trigger Stoke's Vicious Cycle? This is unclear!

The novelty of our study is based on including the evaluation of girls with AIS before any treatment. We cannot exclude a priori that altered brain activations of the sensorimotor network, altered body schema and image, increased attention toward shoulders and back could be a consequence of treatment per se. Physical therapy exercises and brace treatment could increase proprioception and the awareness of the shoulder and back muscles. Our longitudinal study aims to rule out the above-described hypothesis.

Please, see lines 112-118, page 4:

The novelty of our study is also based on including the evaluation of girls with AIS before any treatment. We cannot exclude a priori that altered brain activations of the sensorimotor network, altered body schema and image, increased attention toward shoulders and back could be a consequence of treatment per se. Physical therapy exercises and brace treatment could increase

proprioception and awareness of the shoulder and back muscles. Our longitudinal study aims to rule out the hypothesis described above.

Parallel to this work, we are performing a systematic review on quantitative analyses to study neurophysiological processes in AIS. Most of the studies recruit girls with AIS already in treatment (see Table 1). Results could be affected by attentional and sensorimotor biases due to the physical therapy. Furthermore, there are few studies evaluating subjects both at the beginning and during/after the treatment (see Table 1). Among the longitudinal studies, only 6 recruited subjects who had never undergone any scoliotic treatment.

N° OF STUDIES SINCE 2008	EXCLUSION CRITERIA – SCOLIOTIC TREATMENT				LONGITUDINAL/ NON LONGITUDINAL	
	Postural exercises (%) (a)	Conservative treatment (brace) (%) (b)	Spine surgery (%) (c)	Not specified (%) (d)	Longitudinal studies (%) (e)	Non longitudinal studies (%) (f)
123	15	27	57	43	12	88

Table 1: Evidence from a first screening of the articles selected for the ongoing systematic review. In the first part of the table there are the % of studies that excluded subjects who had already been treated with corrective postural exercises (a), brace (b) and spine surgery (c). Column (d) represents the % of studies which did not specify whether subjects had already undergone scoliosis specific treatment. In columns (e) and (f) there are the % of longitudinal and non longitudinal studies.

P4 – L119: This is interesting! Can the imbalance between the two sensorimotor areas be a biomarker of curve progression? I encourage the authors to state whether they have specific hypotheses. In addition, I am not sure to what groups the authors are referring to as at the beginning of this sentence it is stated that the authors studied static balance in participants with AIS only.

The longitudinal study aims to answer this question.

We would like to understand if the imbalance between the two sensorimotor areas is due to the attentional bias/adherence of the treatment or due to the scoliosis per se. We will compare performances over time and between groups (AIS vs. controls). Up to now, two untreated AIS subjects have undergone the experimental protocol presented in the manuscript. We noticed a different alpha power lateralization between them and subjects already under treatment, recorded during our previous study (Formaggio et al., 2022), (see Figure 1).

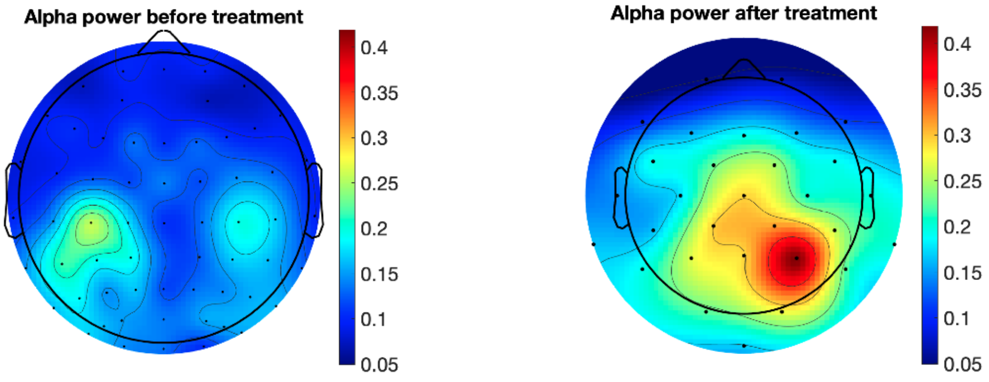

Figure 1: Alpha relative power for two representative AIS girls with right main curve before (topography on the left) and under (topography on the right) treatment. A higher EEG alpha power over sensorimotor areas **contralateral** to the curve is shown in the subject who had not yet undergone treatment. While, an **ipsilateral** lateralization of the alpha relative power is expressed in the subject already under treatment.

References

Formaggio, E., Bertuccelli, M., Rubega, M., Di Marco, R., Cantele, F., Gottardello, F., ... & Masiero, S. (2022). Brain oscillatory activity in adolescent idiopathic scoliosis. *Scientific Reports*, *12*(1), 17266.

P4 – L128: What is the main objective of the functional upper limb tasks. Is it only to induce greater postural challenges? The authors must explain what are the mechanisms that this particular task will permit to study.

We thank the Reviewer for letting us clarify this issue.

The *target reaching* forces the subjects to perform forward trunk movements. It aims to investigate the body schema in adolescent girls with AIS. Possible alterations in the sensorimotor system may modify the planning of the movement within the peripersonal space (i.e., the space immediately surrounding the body).
The aim of the target reaching is now clearly explained:

Please, see lines 90-94, page 3:

The *target reaching* forces the subjects to perform forward trunk movements. It aims to investigate the body schema in adolescent girls with AIS and to determine if alterations in the body schema can modify the planning of movement within the action space immediately surrounding the body, also known as the peripersonal space.

P4 – L132: You reported the range of Cobb's angle for mild and moderate scoliosis above. However, you need to report these ranges here also as it is the section describing the participants and the experimental design.

We thank the Reviewer for the suggestion. We added these details at the beginning of the experimental design paragraph.

Please, see lines 137-140, page 4:

Adolescent girls with a confirmed diagnosis of mild (Cobb angle ranging from 10° to 20°, no spine surgery) and moderate (Cobb angle ranging from 21° to 35°, no spine surgery) scoliosis (AIS) will be enrolled at the Adolescence Spine Diseases Diagnostic and Therapeutic Center of the Padova University Hospital.

P4 – L140: You need to explain the task permitting to assess body schema in static and dynamic conditions.

We thank the Reviewer for letting us clarify this sentence.

The target reaching and the forearm bisection tasks will be applied to evaluate the body schema representational alterations. We corrected the sentence to be clearer. The aim of the target reaching and forearm bisection tasks was clarified in the *Introduction* paragraph.

Please, see lines 147-149, page 5:

At T0, subjects (AIS and CTRL) will undergo two tasks aimed at investigating possible alterations in *body schema* representation.

Please, see lines 85-94, pages 3:

(i) The *forearm bisection task* is a simple and common test widely used in clinical and research fields to investigate the body schema [23]. It requires the subjects to point to the perceived midpoint of their forearm. Since AIS significantly changes the structure of the adolescent spine, thorax, and trunk, we include an innovative task that involves the movement of the trunk. (ii) The *target reaching* forces the subjects to perform forward trunk movements. It aims to investigate the body schema in adolescent girls with AIS and to determine if alterations in the body schema can modify the planning of movement within the action space immediately surrounding the body, also known as the peripersonal space.

P5 – L145: You should report the muscles and the rational for measuring the muscle activities of these muscles. Many previous studies have reported differences in back muscles. Are you going to record muscle activation from different muscles?

We thank the Reviewer for this suggestion.

We plan to record muscle activity from the erector spinae of the back, a muscle that has been extensively studied in the past with reports of asymmetrical muscle activation (Farahpour et al. 2014, 2015), as well as other muscles of the back and trunk (such as rectus abdominis, latissimus dorsi, external and internal oblique) because we aim to investigate possible imbalances and compensatory strategies of muscles involved in forward/lateral bending of the trunk and arm extension (required to perform the target reaching tasks).

We had reported the list of muscles in S1 Appendix, but we briefly mention muscles and rationale in the text as well.

Please see lines 249-252, page 7 :

Before starting the recordings, snap-lead sEMG sensors will be placed bilaterally on the belly of the muscle of the erector spinae, latissimus dorsi, and abdominal muscles (rectus abdominis, abdominal external oblique, abdominal internal oblique) as indicated in S1 Appendix.

Please see lines 339-343, page 9 :

sEMG signals acquired from the trunk and abdominal muscles during the resting state and the target reaching tasks will allow us to quantify a possible imbalance in the muscle activation pattern between the left and right sides of the body, as reported in previous studies.

References

Farahpour, N., Ghasemi, S., Allard, P., & Saba, M. S. (2014). Electromyographic responses of erector spinae and lower limb's muscles to dynamic postural perturbations in patients with adolescent idiopathic scoliosis. *Journal of Electromyography and Kinesiology*, *24*(5), 645-651.

Farahpour, N., Younesian, H., & Bahrpeyma, F. (2015). Electromyographic activity of erector spinae and external oblique muscles during trunk lateral bending and axial rotation in patients with adolescent idiopathic scoliosis and healthy subjects. *Clinical Biomechanics*, *30*(5), 411-417.

P5 – L152: "…during the proposed task…". Explain the task here to help the readers follow you story.

We are sorry for this oversight. During the *resting state* and the *target reaching* we will record signals from the sEMG sensors, wearable IMU devices (accelerometer, magnetometer, and gyroscope), and force plates. Instead, the motion capture system will allow us tracking arm movement during the *target reaching* and *forearm bisection task*. We specified it in the *Foreseen Data Processing* paragraph.

Please see lines 344-346, page 9:

Sensors in the trunk and legs will be used to assess the sway and oscillations of subjects at the level of the upper and lower body, respectively, during resting state and the target reaching.

P5 – L154: "… during tasks." Again, explain the tasks here to help the readers follow you story.

We are sorry for this oversight. We refer the Reviewer to the previous answer (P5 - L152).

Please see lines 346-348, page 9:

Force plates will measure the displacement of the COP, and the motion capture system will allow tracking of arm movement during the target reaching and forearm bisection tasks.

P5 – L158: Will these postural exercises be the same for all participants regardless of the type of scoliosis and the severity of the deformation?

We thank the Reviewer for allowing us to clarify this aspect.

The set of exercises will be thought and adapted from the physiotherapist considering each participant's characteristics and clinical assessment, but all the exercises proposed will share the same aims: to improve postural awareness, proprioception, and ability to dissociate the movements of the lumbar spine from the thoracic one in the different planes of space.

Please, see lines 158-162, page 5:

The set of exercises is believed to improve postural awareness, proprioception, and ability to dissociate the movements of the lumbar spine from the thoracic one in the different planes of space. Treatment will be planned in the individual patient, based on the dysfunction detected in the clinical assessment.

P5 – L171: I am puzzled here as the sampling frequency of the IMU is 142 Hz and this is not a multiple of the sampling frequency of the Vicon which is 1000 Hz. Thus, I am not sure that it is possible to resample up the IMU data to 1000 Hz or to down sample the Vicon data to 142 Hz! Can the authors explain how this is possible?

We apologize for the mistake - IMU data are acquired at 142 Hz only when the IMU system is used as a standalone system. As a matter of fact, we will be using the IMU/EMG system synchronized with the Vicon motion capture system through digital integration, which is already provided and set up by the product developers. When the systems are synchronized through digital integration, IMU and EMG data are acquired directly through the Vicon motion capture software, and they are automatically resampled and synchronized to the motion capture data. As a result, marker data are acquired at 100 Hz, EMG and IMU data are acquired synchronously at 2000 Hz.

Please see lines 170-173, page 5 :

The motion capture system includes a control box (Lock Synch Box, Vicon) to synchronously record analog force plate data (Bertec 4060-10, Bertec Corporation, Columbus, Ohio, United States), IMU and sEMG data (WaveTrack IMU and MiniWave, Cometa srl, Milan, Italy), which will be sampled at 2000 Hz.

P5 – L183: *"Before starting any activity, Ethical clearance will be obtained." This sentence is confusing as below, it is stated that the Ethics committee has approved the research protocol.*

We are sorry for this oversight.

The Ethics Committee approved the experimental protocol on 13 April 2023. We corrected the sentence on line 196.

Please, see line 184, page 6.

Ethical approval has been obtained on April 2023.

P7 – L256: How long will last the resting-state recording? In addition, the authors need to explain how the maximal voluntary contractions will be performed and to measure the MVC of what muscles. Later, there are paragraphs describing the resting-state recording and the measure of the MVC. The authors should consider reorganizing this section as it does not provide valuable information for the resting-state and MVC measures.

We thank the Reviewer for the comments.

The resting-state recording will last 6 minutes (3 min with eyes open, looking at a fixation cross, and 3 min with eyes closed). Guidelines to find the location of the surface electromyography (sEMG) electrodes and instructions to record MVC from each muscle are presented in Table A1 in S1 Appendix. By including these details in the main text, the protocol description would appear long and complex. For this reason we created the S1 Appendix which contains detailed information for reproducing our protocol.

Please see lines 270-273, page 8:

A 6-min EEG resting state recording of quiet upright standing (without shoes) will be acquired in a sound-attenuated room, first with eyes open, looking at a fixation cross (~3 m in front of the subjects), and then with eyes closed.

Please see S1 Appendix, Table A1:

**Muscle**	**Placement**	**MVC**
Latissimus Dorsi (LD)	Over the muscle belly at the T12 level and along a line connecting the most superior point of the posterior axillary fold and the S2 spinous process (Ng et al., 2003, Arch Phys Med Rehabil; Ng et al., 2003, Clin Neurophysiol.; Fan et al., 2014)	Prone lying with the side being tested aligned with the edge of the table. The subject flexes their elbow and extends their humerus parallel to the trunk. Manual resistance is provided at the distal humerus. Stabilization is provided to the ipsilateral scapula and contralateral pelvis. (Park et al., 2013)
Erector Spinae (ES)	Horizontally aligned with the L3-4 interspace, 4 cm lateral to midline (Raschke et al., 1996; Kumar et al., 2010)	Prone lying with the subject's shoulder at the edge of the table and hands at sides. Stabilization is provided at the pelvis and ankles. The subject extends the lumbar spine until the entire trunk is raised from the table (Hislop et al., 2014).
Rectus Abdominis (RA)	Centre of the second uppermost section of the muscle belly (Vera-Garcia et al., 2010)	Supine lying with knees and hips both bent to approximately 90° and hands at sides. Stabilization is provided at the legs, braced by the experimenter, and the thorax is strapped down with a belt. The subject flexes the lower trunk in the sagittal plane against manual resistance provided at the shoulder.

Abdominal External oblique (AEO)	Approximately 3 cm anterior to and mid-way along a line drawn from the lateral pelvic crest to the lateral lower ribcage (Vera-Garcia et al., 2010)	Side lying position with the knees bent and strapped with a belt, and thorax and arms were manually braced by the experimenter. The subject attempts to side bend the upper trunk in the frontal plane while manual resistance is provided.
Abdominal Internal Oblique (AIO)	Halfway between the anterior superior iliac spine of the pelvis and the midline, just superior to the inguinal ligament (Vera-Garcia et al., 2010)	Right and left side bridge position while maximally resisted downward pressure on the pelvis is applied by the experimenter.

References

Fan, J.Z.; Liu, X.; Ni, G.X. Angular velocity affects trunk muscle strength and EMG activation during isokinetic axial rotation. Biamed Res Int. **2014** ,2014, 623191. doi: 10.1155/2014/623191.

Hislop, H.J.; Avers, 0.; Brown, M. Daniels and Worthingham 's Muscle Testing: Techniques of Manual Examination and Performance Testing, 9th ed.; Elsevier: St. Louis, MO, 2014.

Kumar, S. EMG in rotation-flexion of the torso. J Electromyogr Kinesiol. **2010,** 20, 1146-54. doi: 10.1016/j.jelekin.2010.05.003.

Ng, J.K.; Parnianpour, M.; Kippers, V.; Richardson, C.A. Reliability of electromyographic and torque measures during isometric axial rotation exertions of the trunk. Clin Neurophysiol. **2003**, 114, 2355-61. doi: 10.1016/s1388-2457(03)00249-9.

Ng, J.K.; Parnianpour, M.; Richardson C.A.; Kippers, V. Effect of fatigue on torque output and electromyographic measures of trunk muscles during isometric axial rotation. Arch Phys Med Rehabil **2003**, 84, 374-81. doi: I 0.1 053/apmr.2003.50008

Park, S.Y.; Yoo, W.O. Comparison of exercises inducing maximum voluntary isometric contraction for the latissimus dorsi using surface electromyography. J Electromyogr Kinesiol. **2013**, 23, 1106-10. doi: 10.1016/j.jelekin.2013.05.003.

Raschke, U.; Chaffin, D.B. Trunk and hip muscle recruitment in response to external anterior lumbosacral shear and moment loads. Clin Biomech (Bristol, Avon) **1996**, 11, 145-152. doi: 10.1016/0268-0033(95)00044-5.

Vera-Garcia, F.J.; Moreside, J.M.; McGill, S.M. MVC techniques to normalize trunk muscle EMG in healthy women. J Electromyogr Kinesiol. **2010**, 20, 10-6. doi: 10.1016/j.jelekin.2009.03.010.

P7-L263: Why is recording resting-state EEG with and without visual cues important?

We thank the Reviewer for letting us clarify this issue.

We will record EEG resting state data as a baseline static condition. EEG resting state recording of quiet upright standing (without shoes) will be acquired in a sound-attenuated room, first with eyes open, looking at a fixation cross (~ 3 m in front of the subjects), and then with eyes closed. Participants will be instructed to keep their feet positioned parallel and at shoulder width. This is our reference condition, in order to compare the performance of AIS subjects with controls and with their performances during the execution of the tasks. The three major sensory systems (vision, vestibular and somatosensory) are involved in balance and posture, and human balancing during upright stance is more unstable with eyes closed than with eyes open.

Please see lines 269-274, page 8:

Resting-state recording (6 min) will be performed before executing the two tasks as a baseline condition. A 6-min EEG resting state recording of quiet upright standing (without shoes) will be acquired in a sound-attenuated room, first with eyes open, looking at a fixation cross (~3 m in front of the subjects),

and then with eyes closed. Participants were instructed to keep their feet position parallel and at shoulder width.

P7- L266: This is unclear as to estimate the whole body center of mass (COM), one must calculate each segment COM's position. Then, the algebraic sum of all individual COM represents the whole-body COM. It is unclear to me how acceleration and angular velocity of the segments can help calculate the whole-body COM. If I am wrong, I encourage the authors to explain, at least to me, how acceleration and angular velocity signals can be used to estimate whole-body COM.

Thank you for pointing this out.

We made this text confusing when referring to "tasks" and considering in the same sentence the lower limb IMUs, rather than clearly stating that we will measure COM oscillations with the waist-mounted IMU during the static balance task only. During static standing, single waist-mounted inertial sensors are largely used to evaluate balance control and quantify human COM displacement by operating a strapdown integration as originally proposed by Floor et al, 2012 and subsequently validated by Germanotta et al, 2021. The sentence has been revised.

Please see lines 274-277, page 8:

Acceleration and angular velocity data gathered from the IMU placed on the lower back will be processed to estimate COM motion during the standing trial and quantify balance control performance.

References

Floor-Westerdijk MJ, Schepers HM, Veltink PH, van Asseldonk EH, Buurke JH. Use of inertial sensors for ambulatory assessment of center-of-mass displacements during walking. IEEE transactions on biomedical engineering. 2012;59(7):2080–2084

Germanotta M, Mileti I, Conforti I, Del Prete Z, Aprile I, Palermo E. Estimation of human center of mass position through the inertial sensors-based methods in postural tasks: An accuracy evaluation. Sensors. 2021;21(2):601.

P8 – L272: What is sufficient rest?

We thank the Reviewer for letting us clarify this sentence.

We will provide at least 5 min of rest between trials to avoid fatigue, and we will make sure to ask the participant if she may need longer rest time.

Please see lines 281-283, page 8:

5 min of rest between trials will be provided to avoid fatigue accumulation. We will also ask the subject if she may need longer rest time.

P8 -L275: This paragraph should start with a sentence explaining this task's aim and what neural mechanisms this task will permit to study.

We agree with the Reviewer. We specified at the beginning of the sentence the aim of the task.

Please see lines 286-287, page 8:

The task will assess the influence of body schema on the planning of the movement.

P8 – L285: Again, this paragraph should start with a sentence explaining this task's aim and what neural mechanisms this task will permit to study.

We agree with the Reviewer. We specified at the beginning of the sentence the aim of the task.

Please see lines 297-298, page 8:

The task will be used to test the representation of the metric properties of the body.

P9 – L336: What are these dynamic tasks?

We agree with the Reviewer about the misunderstanding of the use of the static and dynamic terms. We replaced the expressions "static task" and "dynamic task" with the specific name of the task we are referring to, as suggested by the Reviewer.

For example, please see lines 339-351, page 9:

sEMG signals acquired from the trunk and abdominal muscles during the *resting state* and the *target reaching* tasks will allow us to quantify a possible imbalance in the muscle activation pattern between the left and right sides of the body, as reported in previous studies. Sensors in the trunk and legs will be used to assess the sway and oscillations of subjects at the level of the upper and lower body, respectively, during *resting state* and the *target reaching*. Force plates will measure the displacement of the COP, and the motion capture system will allow tracking of arm movement during the *target reaching* and *forearm bisection tasks*. In particular, COP data will be used to measure the range of motion in the anterior-posterior (AP) and medial-lateral (ML) directions, the Sway Path length (SP) and the area of the ellipse containing the 95% of the COP trajectory to assess balance during the *resting state* and *target reaching* tasks.

P10 – L364: The authors reported this information above.

We are sorry for this oversight. We removed this redundant sentence from the manuscript.

P10 – L368: Is this sentence necessary?

We removed this sentence from the manuscript.

P10 – L391: Above L183, I understand that the Ethics Committee will evaluate the experimental protocol! Thus, there is some confusion here!

We are sorry for this oversight. The Ethics Committee approved the experimental protocol on 13 April 2023.

Please, see lines 184 and 402-403, pages 6 and 10:

line 184: Ethical approval has been obtained on April 2023.

lines 402-403: Since this experimental protocol has already been approved from the Ethics Committee on April 2023 (n. 5627/AO/22), the study started in June 2023 and lasts two years.
