# Supplementary material for: Experimental protocol to investigate cortical, muscular and body representation alterations in adolescents with idiopathic scoliosis
Source: PLoS One. 2023 Oct 12;18(10):e0292864. doi: 10.1371/journal.pone.0292864 (PMC10569634; doi:10.1371/journal.pone.0292864)
Supplement: Attachment — Submitted filename: Response_to_Reviewers.docx [file pone.0292864.s003.docx]

**Review Comments to the Author**

Reviewer #2: The authors have improved their manuscript, it is now easier to follow the research protocol and the rational for carrying out this study.
I have a minor suggestion and a major concern that need to be addressed by the authors.

We thank the Reviewer for the suggestions. We provide a point-by-point response (text in blue) to each of the comments (text in black). The related changes in the manuscript are in red.

Specifically, we addressed both issues as follows:

Minor suggestion (L65): “… while engaged in a second task (i.e., applying vibration to a muscle tendon…)…”. Electrical vestibular stimulation or tendon vibration are not a secondary task. I suggest rephrasing: "... while either raising their arms with eyes closed or during sensory cues alteration (i.e., ankle tendon vibration or galvanic vestibular stimulation)."

We rephrased as suggested. Please, see page 3 lines 64-67:

Differences between girls with AIS and CTRL have been detected when the experimenters asked the participants to stand upright while either raising their arms with eyes closed or during sensory cues alteration (i.e., ankle tendon vibration or galvanic vestibular stimulation).

Major concern: To assess the kinematics of the center of mass (COM), the authors indicated that they will use data from an IMU located at the waist. This sentence is supported by two references. Looking at the paper of Germanotta et al., (2021), I am surprized that the authors propose to use the strapdown integration to estimate the kinematics of the center of mass. In Figure 6 of the paper of Germanotta et al., (2021), the reported errors (RMSE) between the strapdown integration method and the optoelectronic system are ~ 50 mm and ~ 40 mm/s for postural sway and sway velocity along the anteroposterior axis. The amplitude of these errors is larger for postural sway along the mediolateral axis. Since the calculation of the center of mass requires accurate estimate of the trunk center of mass, it is likely that that these errors will be larger when estimating the center of mass of girls with idiopathic scoliosis, as the estimation of the trunk center of mass is difficult to calculate in person with idiopathic scoliosis.

We thank the Reviewer for having raised this issue, as it allows clarifying this concept.

We agree that to calculate the instant position of the body CoM we need to know the inertial properties and the instant position of each body segment. This can be achieved using a full body marker-set and the optoelectronic system, with subjects’ anthropometry.

However, in literature, the CoM is assumed to be reasonably placed at the iliac spines’ quote and a few centimeters behind the belly button in quiet standing. The rationale for placing an IMU on the lumbar spine as suggested in Germanotta et al 2021, is to make the CoM estimation portable and feasible in clinical settings without calling for ad-hoc laboratories, nor technicians, as the waist-mounted IMU would be close to the CoM and would measure its linear acceleration. The strapdown double integration would then yield an approximation of the CoM instantaneous position.

It is true that when dealing with participants who have axial deformities, such as the idiopathic scoliosis, or performing movements, it may be confusing to consider those measures as representative for the CoM kinematics. In these cases, considering those measures as pelvis motion quantities should be rather preferred.

Moreover, pelvis motion, taken as a parameter to assess balance stability, will be measured using both the optoelectronic system (Vicon) and the IMU. Thus, we will be able to evaluate if the use of the IMU in our experimental protocol is reliable enough to provide quantities to assess subjects’ stability for future clinical applications not demanding for a motion capture lab.

We modified the manuscript accordingly. Please, see page 8 lines 274-280:

Acceleration and angular velocity data gathered from the IMU placed on the lower back will be processed to estimate pelvis motion during the standing trial and quantify participants’ balance control performance [34, 35]. Pelvis kinematics will also be evaluated through the motion capture system. This will allow checking whether the use of a waist-mounted IMU produces reliable quantities to assess AIS subjects stability in clinical settings, without demanding for a motion capture laboratory.
